# Getting control during follow-up visits: the views and experiences of parents on tumor surveillance after their children have completed therapy for rhabdomyosarcoma or Ewing sarcoma

**DOI:** 10.1007/s00520-019-04678-4

**Published:** 2019-02-12

**Authors:** B. Vaarwerk, P. F. Limperg, M. C. Naafs-Wilstra, J. H. M. Merks, M. A. Grootenhuis

**Affiliations:** 1 Department of Paediatric Oncology, Emma Children’s Hospital, Amsterdam UMC, University of Amsterdam, Amsterdam, The Netherlands; 2 Princess Máxima Center for Paediatric Oncology, Lundlaan 6, 3584 CS Utrecht, The Netherlands; 3 Paediatric Psychosocial Department, Emma Children’s Hospital, Amsterdam UMC, University of Amsterdam, Amsterdam, The Netherlands; 4 Childhood Cancer Parent Organization VOKK, Utrecht, The Netherlands

**Keywords:** Childhood cancer, Soft-tissue sarcoma, Treatment completion, Follow-up, Qualitative

## Abstract

**Purpose:**

Patients treated for rhabdomyosarcoma (RMS) or Ewing sarcoma (ES) are subject to extensive follow-up after completion of therapy. The aim of this follow-up is to monitor treatment side effects and to detect relapse in an early phase to improve prognosis after relapse. Little is known about parental emotional experiences during this period. We assessed the views and experiences of parents of children treated for RMS or ES on the follow-up examinations after completion of therapy.

**Methods:**

We conducted two focus group meetings and four semi-structured telephone interviews with parents of children treated for RMS or ES in Dutch pediatric oncology centers. Parents of children 0–5 years after end-of-therapy were invited via letters (response rate 31%) and via social media channels of “Dutch Childhood Association for Children and Parents” (VOKK). An inductive thematic approach was used to analyze the data.

**Results:**

In total, 12 parents (fathers, *n* = 3; mothers, *n* = 9) of 12 patients treated for RMS (*n* = 6) or ES (*n* = 6) participated. Median age at diagnosis for their children was 7.9 years and median time after end-of-treatment was 37 months. Four major themes were identified: content of follow-up, distress and anxiety, search for reassurance and hope, and interaction with others. Parents of children treated for RMS or ES report experiencing significant distress after completion of treatment. They report that their distress was decreased by adequate communication about content, timing, and reasoning behind follow-up.

**Conclusion:**

Physicians should pay attention to the needs of individual parents to reduce distress in the period after completion of therapy.

## Introduction

Over the last decades, the overall survival for pediatric rhabdomyosarcoma (RMS) and pediatric Ewing sarcoma (ES) has increased to around 64% for RMS and 72% for ES [1]. Nevertheless, still many patients experience a tumor relapse after end-of-treatment [2, 3]. Over 50% of the relapses in RMS and ES occur within 2 years from initial diagnosis and survival after relapse in RMS and ES is generally poor [4–6].

After completion of treatment, patients treated for RMS or ES are subject to extensive follow-up. The goal of this follow-up is to detect a tumor relapse before clinical symptoms occur and to monitor treatment side effects, although the clinical value of follow-up after childhood cancer is assessed and debated in several studies [7–9], the views of parents on the content of the follow-up has received no attention. The end-of-therapy entails a major transition in care [10]; it is often a celebrated milestone, but end-of-therapy could also be a period of significant distress and fear of cancer recurrence for parents, especially in the first year [11–14]. Furthermore, parents could also fear long-term sequelae of the treatment and these sequelae could impact the quality of life of patients and parents [15–17]. Besides the fear for long-term sequelae, parents could also experience uncertainty, disease-related fear, and loneliness [18]. Although, in general, elevated levels of distress return to normal over time, the scheduled follow-up examinations could result in additional distress [19]. On the other hand, the routine imaging could give reassurance to parents about the health condition of their child and no follow-up imaging could result in additional distress. Although the coming-off treatment period has been described previously, relatively little is known about fear of recurrence in parents of children treated for RMS and ES.

The results of the studies on the clinical value of follow-up examinations after childhood cancer could result in a change of follow-up recommendations in future study protocols with potential decrease in screening intensity and/or duration. The question arises what do parents need to be in control during the period after completion of therapy. To address this question, we aimed to assess the views and experiences of parents of children treated for RMS or ES on the period after completion of therapy. We asked parents to reflect on their physical and psychological reactions during the follow-up period, what helped them to keep control during this period, and how they reacted to the follow-up examinations. We focused on RMS and ES, since the risk of recurrence in both entities is comparable, survival after recurrence is poor, and the follow-up recommendations for both tumor subtypes are also comparable (see Table 1).

**Table 1:** Follow-up recommendations for patients treated for rhabdomyosarcoma (RMS) or Ewing sarcoma (ES)

Rhabdomyosarcoma*	1st year	2nd year	3rd year	4th year	5th year
Clinical examination	Every 3 months	Every 4 months	Every 4 months	Yearly	Yearly
Imaging primary tumor site	Every 3 months	Every 4 months	Every 4 months	Yearly	Yearly
Lung imaging	Every 3 months	Every 4 months	Every 4 months	Yearly	Yearly
Ewing sarcoma#					
Clinical examination	Every 2 months	Every 2 months	Every 3 months	Every 6 months	Yearly
Imaging primary tumor site	Every 4 months	Every 4 months	Every 6 months	Every 6 months	Yearly
Lung imaging	Every 2 months	Every 2 months	Every 3 months	Every 6 months	Yearly

*Follow-up recommendations according to the European *pediatric* soft-tissue sarcoma Study Group-RMS-2005 protocol for localized disease

#Follow-up recommendations according to the EWING 2008 protocol

## Methods

### Study design

To assess the views of parents on the follow-up examinations after completion of therapy, we conducted a qualitative analysis with focus group (FG) meetings and semi-structured telephone interviews. We chose to use FG meetings to obtain a broad overview of the views and experiences of the group of parents on the follow-up after completion of treatment. A FG can provide more detailed information about an experience compared to a questionnaire and generates more disclosures or discussion compared to individual interviews. This study was conducted in the Academic Medical Centre (AMC) Amsterdam between January 2017 and December 2017. We invited parents of patients treated for RMS and ES in their follow-up period (0–5 years after completion of therapy) and in persistent remission of their disease to participate in the FG meetings. We recruited parents of children treated at the AMC and, to include a more diverse group of parents, we also recruited parents from other regions via the Dutch *Childhood Cancer Parent Organization* (VOKK). Parents were eligible if their child was 0–18 years at time of diagnosis of RMS or ES.

The FG meetings were held at the AMC and at the office of the VOKK. Semi-structured telephone interviews were conducted with parents who were not able or who were not willing to participate in the FG meetings but were willing to share their experiences.

In total, 26 parents of children treated in the AMC were invited by letter, with a reminder after 2 weeks; 11 parents responded and finally, 8 parents participated in this study (response rate 31%). Furthermore, we invited parents via the newsletter and social media channels (such as Facebook) of the VOKK, resulting in 4 additional participants. The institutional review board of the AMC decided that the Medical Research Involving Human Subjects Act (WMO) did not apply for this study.

### Data collection

The FG meetings were moderated by two researchers (B.V. and M.A.G.) and lasted 1.5–2 h; both were unacquainted with participating parents. A topic guide was developed for the FG meetings based on a review of the literature on adult patients and input from pediatric oncologists (Table 2). The topic guide was designed to determine the views of parents on the period after completion of therapy, to determine physical and psychological functioning experienced by parents in relation to the follow-up visits, and to assess whether follow-up imaging influenced their functioning in everyday life. Furthermore, it was evaluated if questions were open and in line with the research question. The focus group discussions were audio recorded (with the permission of the participants). The semi-structured interviews were conducted by one researcher (B.V.). The same topic list was used for these interviews and the interviews were audio recorded.

**Table 2:** Topic list used in the FG meetings and in the semi-structured interviews

Opening questions	
- Could you introduce yourself, your family situation, and elaborate on your child’s illness?	
- What does the follow-up of your child look like at the moment, in terms of frequency and content of the follow-up?	
Key questions	
- How did you experience the moment directly after end-of-treatment?	
- How do you experience the moments of follow-up appointments?	
- How do you experience the follow-up imaging?	
- Do you experience specific emotions and/or stress?	
- What helped you to control your emotions around a follow-up visit?	
- What is the influence of the following factors on emotions/stress;	
- Upcoming follow-up appointments?	
- Health status of child?	
- Partner or other family members?	
- What is the influence on your daily life functioning?	
Final question	
- Do you have specific recommendations for future follow-up	

### Data analysis

The FG meetings and interviews were audio recorded and transcribed verbatim. These data were analyzed by using an inductive thematic approach, suitable to report a range of experiences [20]; three authors (B.V., P.F.L., M.A.G.) were involved in the analysis.

Data analysis was performed by using MAXQDA software (version 12.2.1.) First, the researchers independently read, re-read, and subsequently open-coded the transcripts of the FG meeting by highlighting and categorizing keywords. Thematic analysis was used to identify recurring topics and these general codes were discussed in team meetings and grouped into themes. No formal interrater reliability assessment was done.

In addition, the semi-structured interviews were read and coded by one author (B.V.). All themes were reviewed and, if necessary, adapted, after coding of a new interview. This process continued until data saturation was reached. No new themes emerged following the semi-structured interviews.

## Results

In total, 12 parents (9 mothers, 3 fathers) of 12 patients treated for RMS (*n* = 6) or ES (*n* = 6) participated in this study. In both FG meetings, four different parents participated and the other four parents participated in semi-structured interviews. The median age at diagnosis for the children of participating parents was 7.9 years (range 0.5–15.5 years) and the median time since end-of-treatment was 37 months (range 5 to 52 months).

Four major themes were identified, discussed in detail below. The themes covered the content of follow-up, distress and anxiety in the period after completion of therapy, search for reassurance and hope, and interaction with others in the period after completion of therapy. Table 3 describes the major themes and corresponding examples of statements of the participants.

**Table 3:** Themes emerging from focus groups and semi-structured interviews

Themes	Characteristics*	Examples
Content of the follow-up	Mother, RMS, 26 months	“The period directly after end-of-therapy felt like I had to swim, but I did not know how to do it”
	Mother, ES, 50 months	“The follow-up period is tough but necessary”
	Mother, ES, 52 months	“We always felt relieved after the MRI, since our son always gets very upset by the anaesthesia”
	Mother, ES, 51 months	“Besides the follow-up imaging and the appointment with the paediatric oncologist, we also have appointments with the orthopaedic surgeon, urologist and rehabilitation physician.”
	Mother, ES, 38 months	“I am surprised that the follow-up is different between hospitals”
	Mother, ES, 52 months	“If the protocol prescribes the end of follow-up, than it is okay for me”
Distress/anxiety over time	Mother, RMS, 35 months	“The first year, I was getting nervous a month before the follow-up”
	Mother, ES, 50 months	“You just want the five years to get over”
	Father, RMS, 29 months	“On the day of the imaging I am always more agitated”
	Mother, RMS, 12 months	“You get more confident over the years”
	Father, ES, 50 months	“Especially the first few times I was really anxious”
	Mother, ES, 51 months	“It would be nice if the different specialists would also discuss their individual advice with each other”
	Mother, ES, 52 months	“I know that the outpatient clinic from our oncologist is open on Monday and Friday, so we always arrange the imaging on Friday to have the results on Monday.”
Search for reassurance and hope	Mother, RMS, 47 months	One mother on the value of the MRI: “it feels reassuring to know that everything looks good on the inside”
	Father, ES, 50 months	“It is unbelievable how strong our boy was, which also made us feel strong and proud”
	Father, RMS, 5 months	“You do get the information one way or another, because during follow-up you also receive unsolicited information from parents sitting next to you”
	Mother, RMS, 12 months	“Our oncologist tells us that the risk of recurrence is small after the first year, but what is small? I cannot find stories of children surviving this tumor on the internet, so where are these survivors?”
Interactions with others	Mother, RMS, 35 months	“I notice that I want to protect my child in everything and I need to be aware not to do this too much”
	Mother, RMS, 26 months	“On the day of a follow-up visit our other children are really nervous, so when we get the results we call them immediately which make them really happy.”
	Mother, ES, 50 months	“My husband always says ‘it not useful to worry about something that is not there and may never be there’. This really helps me as well.”
	Mother, RMS, 47 months	“Before, when I heard a mother talking about her child having the flu, I thought, let us swap our situation … now I am able to react with compassion again.”

*Characteristics include sex of parent, diagnosis of child, and time since end-of-treatment. *ES*, Ewing sarcoma; *RMS*, rhabdomyosarcoma

### Content of follow-up

The follow-up for the children of participating parents was depending on the type of treatment, type, and localization of the tumor, but also on the suggested follow-up regimens of the different hospitals. Most parents describe the moment directly after end-of-treatment as a very difficult period. Although the treatment was finished, they did not feel relieved. A mother described this period as “it felt like I had to swim, but I didn’t know how to do it” (diagnosis: RMS, time since end-of-treatment: 26 months)*.*

For all children, the follow-up consisted of regular imaging, with extension of the interval over time. One parent described the follow-up moments as “tough but necessary” (mother, ES, 50 months), and parents with younger children expressed that the necessity of general anesthesia during follow-up made them extra nervous.

How parents experienced the different parts and content of follow-up was related to previous experiences during treatment and follow-up and potential adverse effects experienced by the child. For example, a mother of a child treated for ES reported the follow-up visits to the orthopedic surgeon as most stressful, because in multiple occasions, the visit to the orthopedic surgeon led to additional surgery. For other parents receiving the results of the MRI was most stressful, because the initial diagnosis was also confirmed on MRI results.

Most parents were aware of the content of the follow-up prescribed in the treatment protocol, which was also explained by their pediatric oncologist. In general, parents understood that the extension of the interval between the imaging was possible because of the decreasing chance of relapse over time. To the question whether parents would accept it if in the future no imaging would be performed, one mother replied; “the follow-up is according to a protocol and is explained by the oncologist and because of the explanations I felt confident that this is okay; however, the moment approaches that the follow-up imaging might no longer be done and that feels very difficult” (ES, 52 months). Another mother said “oh I don’t know, I hope that moment doesn’t come soon”(ES, 15 months).

### Distress and anxiety over time

The most reported theme in this study was distress and anxiety after end-of-therapy; almost all parents reported experiencing distress during the follow-up period and this was influenced by several factors. Most parents reported experiences of distress in the years after end-of-therapy, increasing in the days (for one mother even a month) prior to follow-up imaging; some parents experienced physical complaints, others were agitated prior to follow-up. Several parents described feeling extremely tired after having received the positive results of the follow-up visit.

Although most parents described the follow-up as stressful, some reported experiencing it as pleasant to be back in the hospital. One mother described it as “feeling like coming home” (RMS, 47 months), whereas another mother preferred to stay home waiting for a call from her partner (ES, 52 months).

The distress was influenced by the time passed since end-of-therapy; although most parents felt relieved when the imaging did not show signs of tumor recurrence, this feeling did not endure for a long time in the first year of follow-up. Over the years, the distress decreased and the extension of the interval between follow-up decreased the feeling of distress for a longer period of time.

Distress was further influenced by treatment-related adverse events, such as fatigue and physical rehabilitation of the child. Several parents described that they could have never imagined it would take years for their child to fully rehabilitate after end-of-treatment.

Distress during the follow-up period further accumulated by inadequate communication between different medical specialists. For example, one mother told that her daughter (ES, 51 months) had regular follow-up visits with a rehabilitation physician and with an orthopedic surgeon; however, both physicians gave contrary advice without consulting each other which caused distress and uncertainty for the child.

Communication regarding the results of follow-up examinations also influenced distress. All parents made arrangements with their oncologist regarding the communication of the results; this structure helped them to reduce distress around the follow-up, as illustrated by one mother; “I know that the outpatient clinic from our oncologist is open on Monday and Friday, so we always arrange the imaging on Friday to have the results on Monday.” ( mother, ES, 52 months).

Increasing age of the children also influenced distress; some parents noticed that their children were aware or were becoming aware of their history of cancer. These children were also nervous before follow-up imaging or very vigilant about their own health. Some children were examining their body for potential signs of relapse regularly.

Participating parents also reported positive consequences of the disease such as developing a different attitude towards life. One mother described that “it does have positive sides; our family, my husband and I, became much closer” (ES, 51 months). Another mother said “because of what has happened, I’m nowadays more aware of the simple things in life; I could sit at a table and just enjoy being there” (RMS, 12 months).

### Reassurance and hope

To cope with the distress around the follow-up examinations, many parents were looking for reassurance and hope and had their own strategies of coping. Most parents reported to find reassurance in the positive results of the follow-up imaging. Parents described it as “reassuring to know that everything looks good on the inside,” although they were aware that an MRI was not predictive for the upcoming period.

Previous experiences determined the reassurance. One mother reported that, although she was anxious for the results of the MRI, she did not worry about the result of the chest X-ray since her child (ES, 38 months) did not have lung metastases at diagnosis.

Parents felt reassured by (improvements in) the health condition of their child and also by their strength, attributing positive characteristics to the child. One father said “it is unbelievable how strong our boy was, which also made us feel strong and proud” (ES, 50 months)*.*

Furthermore, hope of parents was influenced by information received during the follow-up period, but also information received during treatment. Some parents were actively looking for “all” available information; others were trying to protect themselves by not looking for additional information, although they did receive information passively. A participating father explained “you do get information one way or another, because during follow-up you also receive unsolicited information from parents sitting next to you” (RMS, 5 months). Parents specifically mentioned survival chances; almost all parents were aware of survival chances; nevertheless, they reported to find it difficult to understand the meaning of risks. One mother described her feelings about risks of relapse as follows: “Our oncologist tells us that the risk of relapse is small after the first year, but what is small? I can’t find stories of children surviving this tumour on the internet, so where are these survivors?” (RMS, 12 months).

### Interaction with others

The period after end-of-treatment influenced the interaction with partners, especially around the follow-up visits. Some parents discussed their feelings with their partners, whereas others did not discuss their feelings at all, which sometimes led to tension in their relationship/marriage. Mothers often indicated that fathers were more sensible or less emotional with respect to the follow-up visits. The follow-up visits also influenced other children in the family. One mother stated that “On the day of a follow-up visit our other children are really nervous, so when we get the results we call them immediately which made them really happy” (RMS, 26 months)*.*

Parents described that they had the feeling that their friends and relatives did not understand their situation; their friends and relatives generally thought that parents should feel relieved since the treatment was finished, whereas parents had the feeling they were still in the middle of the whole process. This feeling also faded out, as one mother described “Before, when I heard a mother talking about her child having the flu, I thought, let’s swap our situation … now I’m able to react with compassion again” (RMS, 47 months)*.*

## Discussion

This qualitative study describes the views and experiences of parents of children treated for RMS or ES on the follow-up examinations after completion of therapy. We asked parents to reflect on their physical and psychological reactions during the follow-up period, what helped them to keep control during this period, and how they reacted to the follow-up examinations. The results centered around four major themes; the content of follow-up, distress and anxiety in the period after completion of therapy, search for reassurance and hope, and interaction with others in the period after completion of therapy. This study helped us in our understanding what parents need to feel in control during the period after completion of treatment.

This period is difficult for parents; it entails a major transition in pediatric oncology care and can cause significant distress [10–14]. Whereas social support is generally high at time of diagnosis, support tends to decline over time [21]. Although the treatment has finished, the threat of a potential relapse becomes apparent in parents [19, 22]. During this period, parents and child try to reintegrate in everyday life, while children might still suffer from adverse effects caused by the treatment. Distress and anxiety caused by fear of cancer recurrence play a significant role during the follow-up period, which is traditionally described as *The Damocles Syndrome* [23]. Fear of cancer recurrence can significantly impact the quality of life of cancer survivors, which was shown in other cancer types [24].

Because of the qualitative nature of this study, we were able to obtain a detailed description of the period after completion of therapy. Most participating parents felt reassured by the scheduled follow-up examinations; nevertheless, these examinations also evoked additional distress and anxiety, which was reported previously [19].

However, the experienced distress and anxiety were not only caused by fear of cancer recurrence, but also by treatment-related adverse effects, which are common in patients treated for RMS or ES [25]. These adverse events depend on the tumor localizations, received treatment, and on patient characteristics. Therefore, parents indicated that specific parts of follow-up visits, for example, visits to the orthopedic surgeon, were more important than other parts.

Throughout the follow-up period, parents were continuously looking for reassurance and hope. Reassurance was found in the radiologic examinations and was further enhanced by getting control over specific situations, for example, by making strict arrangements around the follow-up examinations. These strategies can be considered cognitive control coping strategies to get a hold on the situation, which is for the most part uncontrollable [26]. This study shows again the importance of coping strategies throughout the cancer trajectory. Many studies have shown that psychological functioning of both children and parents is affected by how families cope with the illness [27, 28]. It is important that health care providers are sensitive to the control strategies used by parents and take this into account during the follow-up process. Health care providers could play a significant role in promoting normal family life by providing clear information on the condition of the child and enhancing family coping strategies [29].

Participating parents felt reassured by the knowledge that the risk of relapse decreased over time and with that also the frequency of follow-up; however, some parents experienced more distress by knowing the risk of relapse specifically. Health care providers need to think of which information at which time point is given to individual parents [30, 31].

Finally, the period after completion of therapy affected the interaction with partners, other children, and their social life. Although parents reported, on the one hand, to enjoy life more, they also reported feelings of loneliness. As previously suggested by Kearney et al., parents need to receive early and ongoing assessment of their mental health needs with access to appropriate interventions to optimize parents well-being but also family functioning [32].

### Limitations

A limitation of this study is that the response to the invitation letters was only 31% and most of the participating parents were mothers. Underrepresentation of fathers in studies on the impact of the off-therapy period after childhood cancer treatment was previously reported and the results of this study indicate that there might be differences in views and coping styles between fathers and mothers [13]. Furthermore, we only included Dutch parents making it difficult to determine whether our results are generalizable to families with other backgrounds. A further limitation is that we included patients treated for RMS or ES. We are uncertain whether the views of parents on the follow-up examinations after end-of-therapy are comparable since specific patients may experience specific adverse effects. Nevertheless, major themes identified in this study are comparable to those identified in a review focused on the psychosocial impact of childhood cancer treatment including studies from different countries and patients with different tumor types [13]. Therefore, we believe that the views of parents of children treated in other countries and with different tumor types might be comparable.

Furthermore, although the qualitative design of the current study enabled the collection of detailed data, it is difficult to assess whether the participating parents were representative for the total group of parents. We tried to limit selection bias by inviting parents via letters and via the VOKK website and social media channels. We conducted semi-structured interviews with parents not able or not willing to participate in FG meetings; however, the number of interviews was small (*n* = 4).

### Clinical implications

Our findings are of utmost importance for clinical practices to be acted upon by health care providers. We would advise physicians to pay attention to the individual needs of parents to reduce the distress in the off-therapy period and to focus on parental coping strategies. Future studies and education should focus on communication strategies to discuss follow-up care with parents, to assist parents in the follow-up period [33]. Furthermore, continuing attention should be paid to the mental health needs of parents also after completion of therapy.

In light of the recent and ongoing studies regarding the clinical value of follow-up imaging after end-of-therapy, future follow-up recommendations should be adapted and more tailored on tumor characteristics, but they should also take parental preferences into account [8, 34, 35].
